# MR enterography to evaluate sub-clinical intestinal inflammation in children with spondyloarthritis

**DOI:** 10.1186/1546-0096-10-6

**Published:** 2012-02-08

**Authors:** Matthew L Stoll, Ashish S Patel, Marilynn Punaro, Molly Dempsey-Robertson

**Affiliations:** 1 UT Southwestern Medical Center/Department of Pediatrics/5323 Harry Hines Boulevard/Dallas, TX 75390-9063, USA; 2 Texas Scottish Rite Hospital for Children/Department of Rheumatology/2222 Welborn Street/Dallas, TX 75219, USA; 3 Dr. Stoll: University of Alabama at Birmingham/Department of Pediatrics/Children's Park Place/1601 4th Ave S./Suite 210/Birmingham, AL 35233, USA; 4 Texas Scottish Rite Hospital for Children/Department of Radiology/2222 Welborn Street/Dallas, TX 75219, USA

**Keywords:** Spondyloarthritis, Juvenile idiopathic arthritis, Inflammatory bowel disease, MRI

## Abstract

**Background:**

Magnetic resonance enterography (MRE) is an established tool to evaluate for changes associated with inflammatory bowel disease (IBD), but has not been studied in sub-clinical IBD. We sought to evaluate the use of MRE in children with spondyloarthritis (SpA), who are at risk of having sub-clinical gut inflammation.

**Methods:**

Children with juvenile idiopathic arthritis (JIA) with evidence of intestinal inflammation as evidence by an abnormal fecal calprotectin assay were offered MRE of their intestines. Flavored sports drink containing polyethylene glycol 3350 was used as oral contrast. Glucagon was used to arrest peristalsis. Patients were imaged in the prone position on a 1.5 T scanner. Heavily T2-weighted fat-suppressed coronal and axial images using breath-hold technique were obtained, followed by post-gadolinium fat-suppressed T1-weighted gradient echo images.

**Results:**

We recruited five children with juvenile idiopathic arthritis (JIA); four had SpA, and one had poly-articular JIA. All five had evidence of intestinal inflammation based upon a positive fecal calprotectin assay and successfully completed the MRE. Three of the studies showed findings suggestive of IBD, including thickening and contrast uptake at the terminal ileum (TI) in one child, contrast uptake of the distal ileum in another, and prominent vasa recta and mesenteric lymph nodes in the third. The child with evidence of inflammatory changes at the TI underwent colonoscopy, which revealed inflammatory bowel disease limited to the TI.

**Conclusions:**

MRE can be used to evaluate for subclinical IBD in children with JIA. This protocol was safe and well-tolerated, and identified mild changes in three of the subjects.

## Background

Approximately two-thirds of adults with spondyloarthritis (SpA) have inflammatory intestinal changes similar to those detected in inflammatory bowel disease (IBD) [1]. Similar findings were reported in a small pediatric study [2]. However, these studies used colonoscopy, an expensive and invasive tool and thus one that is not well suited for research studies. Studies using barium swallow and sigmoidoscopy have identified sub-clinical intestinal inflammation in lower percentages of SpA patients, suggesting decreased sensitivity in that population [3,4]. Computed tomography involves significant amounts of radiation exposure, and ultrasound is limited in some centers by operator-dependence [5]. However, one potential tool that could be used safely to evaluate the intestines in children and adults with SpA is magnetic resonance enterography (MRE).

MRE is an accepted tool to diagnose and monitor IBD. Although it does not visualize early mucosal changes such as aphthous ulcerations, MRE allows for the detection of bowel wall thickening and enhancement, as well as extramural complications of IBD, including strictures, fistulas, sinus tracts, abscesses, fibro-fatty proliferation, and lymphadenopathy [5-10]. Studies in adults and children have shown MRE to be accurate in the diagnosis of IBD, distinguishing it from other causes of abdominal pain with sensitivity 82 - 96% and specificity > 90% [11-15].

These studies raise the possibility that MRE may be of benefit to screen for subclinical intestinal inflammation in SpA patients. We previously recruited children with enthesitis-related arthritis (ERA) and other subtypes of juvenile idiopathic arthritis (JIA), and obtained measurements of fecal calprotectin, a stool study that assesses the presence of inflammation based on neutrophil-derived proteins that are resistant to metabolic breakdown by intestinal bacteria and can assist in differentiating inflammatory from non- inflammatory states [16]. In that study, we showed elevated fecal calprotectin levels in ERA patients, as compared to children with other JIA subtypes, as well as controls consisting of children with unrelated connective tissue diseases and non-inflammatory causes of joint pain [17]. A limitation of fecal calprotectin is that it does not provide any information as to the location of the inflammation or the presence of specific complications potentially associated with IBD. Thus, to evaluate the anatomic location and extent of sub-clinical intestinal inflammation in children potentially at higher risk of intestinal inflammation, we performed a sub-study of the above, offering MRE to JIA patients with elevated fecal calprotectin levels.

## Methods

### Patients

This was a prospective sub-study of fecal calprotectin levels among patients with JIA [17], diagnosed according to the International League of Associations for Rheumatology (ILAR) criteria [18]. Calprotectin levels were measured via ELISA in a commercial laboratory (ARUP, Salt Lake City, UT), with values < 50 micrograms/gm considered negative, 50 - 120 borderline, and ≥ 121 elevated. Inclusion criteria for the current study were a fecal calprotectin level of at least 121 micrograms/gm obtained as part of that study. Exclusion criteria were inability to cooperate with the procedure, allergy to IV contrast, renal insufficiency, MRI incompatible devices or implants, and pregnancy; in practice, the only exclusion criteria applied was inability to undergo MRI without sedation. There was no strict age cut-off, although most children under age 8 or 9 would not be expected to be able to undergo unsedated MRI. All of the JIA patients with elevated fecal calprotectin levels (≥ 121 micrograms/gm) who were potentially mature enough to undergo MRI without sedation were invited to do so; of the 8 who met the inclusion criteria, 5 agreed to participate. This study was approved by the Institutional Review Board at the UT Southwestern Medical Center. Informed consent was obtained from each subject's legal guardian, and assent was obtained from each child.

### MR enterography

#### Preparation

Patients were NPO for six hours prior to the study. We used one packet (17 gm) of polyethylene glycol 3350 (over the counter Miralax^®^) dissolved in a flavored commercial sports beverage (Gatorade^®^) in order to increase bowel wall distension. Over the course of 2.5 hours, they were given a total volume of 1250 ml; one quarter of the total volume was taken approximately every 30 minutes, with the final dose given 15 - 30 minutes prior to the study. To inhibit bowel peristalsis, patients were administered 0.5 mg glucagon IV at the onset of the study; a second dose was given if the radiologist (MDR) determined that there was motion artifact suggestive of peristalsis.

#### MR examination

Patients were imaged in the prone position. Utilizing a body surface coil an MRI exam was acquired on a GE 1.5 Tesla (Milwaukee, WI) MR scanner. To minimize motion artifact, the patient was asked to hold his or her breath during image acquisition, and as stated above, bowel motion was reduced with the administration of glucagon. Sequences and parameters are summarized in Table 1. The precontrast images allowed visualization of bowel wall thickening, mesenteric lymph nodes, and prominent vasa recta. IV gadolinium, 0.1 mmole/kg (max 10 mmole) of gadoteridol (Prohance; Bracco diagnostics) was then administered, and additional images were acquired for the detection of bowel enhancement suggesting active inflammation.

**Table 1:** MRI sequences used

Type	Parameters
**Pre-contrast**	
Coronal SSFSE T2w	TR minimum ~750, TE 70 1nex, bw 83.3 320x 224, asset, zip 512, 5/0
FS Coronal 2D FIESTA	TR 6.8, TE 2 (minimum full),1 nex, bw 83.3, flip angle 50, 288 × 256, phase fov 0.9, asset, zip 512,, 5/0 cor 6/0 axial
FS Axial 2D FIESTA	Same as above
**Post-contrast**	
FS Axial 2D fSPGR	TR 245, TE minimum, fa 70, bw 83.33, nex 1, 320 × 160, phase fov 0.8, 5/0 cor, 6/0 axial asset
FS Coronal 2D fSPGR	Same as above
Coronal 3D LAVA	TR 4.2, TE 2, TI 7, fa 12, bw 62.5, 5 mm 30 locs/slab, 288x 192, asset, zip 2, fat sat

MRI sequences used; the length of each of the sequences ranged from 12 - 45 seconds. FIESTA = fast imaging employing steady state acquisition, FS = fat-suppressed, fSPGR = fast spoiled gradient recalled echo, LAVA = liver acquisition with volume acceleration, SSFSE = single shot fast spin echo, T2w = T2-weighted

## Results and discussion

### Patient population

Nine subjects with JIA had elevated fecal calprotectin levels. One was incapable of undergoing unsedated MRI, and three declined to participate in the MRI study. Thus, five patients agreed to participate, and all five completed the study (Table 2). There were no obvious differences between active joint count, disease duration, or presence vs absence of gastroenterology symptoms between the five who participated and the three who did not (data not shown.) All five had JIA and had an elevated fecal calprotectin level (median 249). Four had ERA; one had poly-articular JIA. Only one (patient # 4) had significant gastrointestinal symptoms, consisting of abdominal pain and weight loss prior to treatment with corticosteroids, although he was asymptomatic at the time of the study.

**Table 2:** Study patients

Patient #	Dx	Age/sex	Disease duration (months)	Fecal calprotectin*	Medication use
1	ERA	10.8/M	15	231	MTX, ETA
2	ERA	16.1/M	37	249	None
3	pJIA	11.5/F	118	678	NSAID, MTX
4	ERA	10.9/M	11	310	NSAID, PRED, MTX
5	ERA	14.1/M	12	171	ETA

* Micrograms/gm of stool. Normal < 121. Abbreviations: ERA = enthesitis-related arthritis, ETA = etanercept, MTX = methotrexate, NSAID = non-steroidal anti-inflammatory drugs, pJIA = poly-articular juvenile idiopathic arthritis, PRED = prednisone.

### MR enterography findings

**Figure 1:**
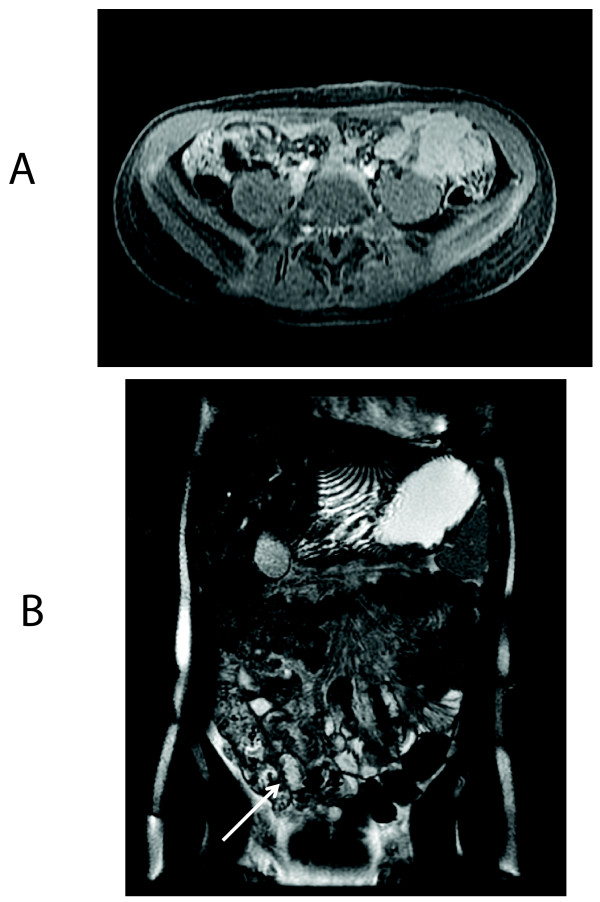
**Normal bowel**. Axial T1 fSPGR with fat saturation post contrast at the area of the distal ileum in 16 yo male with ERA (patient 2); no bowel thickening or contrast uptake is evident **a**. Coronal 2D FIESTA (pre-contrast image) in 11 yo female with poly-articular JIA, with a normal TI indicated by the arrow (patient 3) **b**.

**Figure 2:**
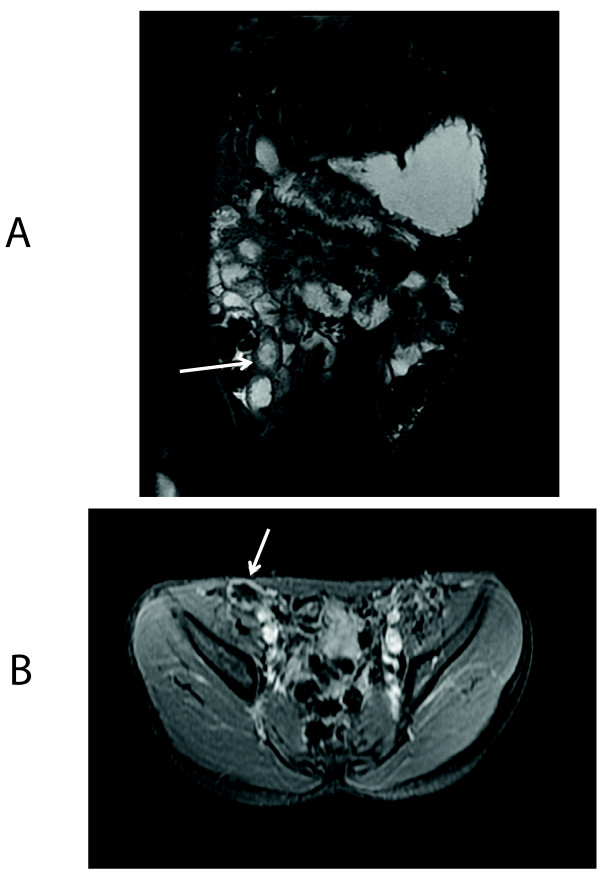
**10 yo male with ERA (patient 1)**. Coronal 2D FIESTA with fat saturation (pre-contrast) showing thickening at the TI (arrow) **a**. Axial T1 fSPGR with fat saturation post-contrast showing enhancement at the terminal ileum (arrow) **b**.

**Figure 3:**
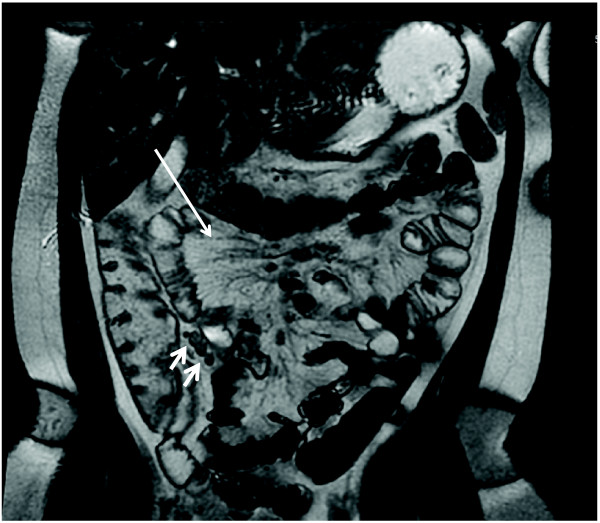
**Another 10 yo male with ERA (Patient 4)**. Coronal 2D FIESTA (pre-contrast) showing prominent vasa recta (large arrow) and mesenteric lymph nodes (small arrows). The TI is not visualized on this sequence, and was normal in this patient (not shown).

**Figure 4:**
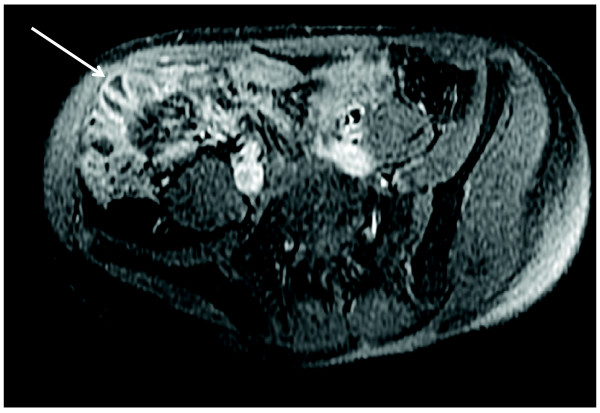
**14 yo male with ERA (Patient 5)**. Axial T1 fSPGR with fat saturation post contrast showing abnormal enhancement at the distal ileum (arrow). There was no obvious bowel wall thickening.

Patients # 2 and 3 had normal studies (Figure 1.) Patient 1 revealed increased bowel wall thickness (4 mm; Figure 2A) and enhancement (2B) at the terminal ileum (TI). Patient 4 showed multiple mesenteric lymph nodes and prominent vasa recta (Figure 3.) Patient 5 showed increased contrast uptake at the distal ileum, without thickening (Figure 4.) There was no obvious correlation between the presence or type of bowel wall inflammation and the fecal calprotectin levels.

### Safety

The study was tolerated without any serious adverse events. Patient 1 had mild emesis after the second dose of glucagon. No other adverse events were reported.

### Patient follow-up

The patients were followed for a median of 9.6 months (range 5.3 - 15) after the MRI. Patient 1 subsequently developed abdominal pain and was therefore referred to gastroenterology; 5 weeks after the MRE, he underwent colonoscopy, which revealed non-specific inflammatory changes limited to the TI, prompting a diagnosis of IBD. His medical management was changed from etanercept to adalimumab, in order to treat his underlying bowel disease; he subsequently has had improvement in arthritis, albeit still active at the final visit. Patient # 4 was referred to gastroenterology but never made the appointment. Nevertheless, due to the presence of active arthritis as well as acute anterior uveitis, he was also started on adalimumab, with improvement in his arthritis symptoms, but continued to have active arthritis at the end of the follow-up period. None of the other patients underwent changes in their medical management and were doing well at the final follow-up. Patients 2, 3, and 5 were not diagnosed with IBD or other intestinal illnesses.

The primary implication of this study is in demonstrating that MRE may be a tool with which investigators can evaluate for subtle inflammatory changes in patients with SpA. The gold standard, ileocolonoscopy, is invasive and expensive, and thus not well-suited for research purposes. Fecal calprotectin levels are elevated in children with SpA [17], but they do not provide specific anatomical information within the intestines, and it appears that their levels can be increased nearly two-fold by use of non-steroidal anti-inflammatory drugs [19,20]. Likewise, wireless capsule endoscopy (WCE) can identify subclinical changes in SpA patients [21]; however, WCE does not identify changes beyond the intestinal mucosa, and is limited by risk of obstruction.

This study also provides further exploration of the connection between intestinal inflammation and SpA. We have previously hypothesized that in children with SpA, intestinal inflammation helps maintain peripheral synovitis via mechanisms yet unclear [22]. However, the extent of inflammation need not be extensive; indeed, while the majority of patients with SpA have intestinal inflammation [1], only a minority develop frank IBD. Likewise, in our study, the MRI findings were subtle, and none showed the extensive complications previously reported in IBD patients [9,10], and thus none of the patients were diagnosed with IBD on the basis of the MRI. Tumor necrosis factor (TNF) inhibitors differ in their capacity to treat established IBD, with etanercept less efficacious as compared to some of the TNF monoclonal antagonists [23,24]. It follows that they may also differ in their capacity to treat sub-clinical intestinal inflammation, such as that identified in this study. If it is indeed the case that sub-clinical intestinal inflammation helps maintain peripheral arthritis in SpA, long-lasting remission may not be possible in the absence of control of this intestinal inflammation. Indeed, registry data indicates that successful withdrawal of etanercept in children with ERA is rare [25].

This study has several limitations. The sample size was small, there was no control population, and there was no gold standard study performed. Nevertheless, we believe that these findings are specific and meaningful; two of the patients had changes in the distal ileum, a common site of inflammation in IBD [26], and in one, colonoscopy confirmed inflammation at that location.

## Conclusions

Magnetic resonance enterography identified subclinical intestinal inflammation in three children with spondyloarthritis. Future studies should prospectively screen newly-diagnosed children with ERA for intestinal inflammation with tools such as fecal calprotectin or MRE and evaluate whether or not the presence of such inflammation predicts response to anti-TNF therapy or ability to be withdrawn successfully from such therapy.

## List of abbreviations used

ERA: enthesitis-related arthritis; FIESTA: fast imaging employing steady state acquisition; fSPRG: fast spoiled gradient recalled echo; IBD: inflammatory bowel disease; JIA: juvenile idiopathic arthritis; LAVA: liver acquisition with volume acceleration; MRE: magnetic resonance enterography; SpA: spondyloarthritis; TI: terminal ileum.

## Competing interests

The authors declare that they have no competing interests.

## Authors' contributions

MS - study design, patient recruitment, manuscript preparation; AP - data analysis, manuscript review; MP - study design; MDR - study design, interpretation of MR images, manuscript preparation. All authors read and approved the final manuscript.
